# Age-dependent changes in plasma concentrations of 25-hydroxyvitamin D may complicate vitamin D status assessment of immature cats

**DOI:** 10.3389/fvets.2024.1365204

**Published:** 2024-05-02

**Authors:** Robert C. Backus, Devon C. Ueda

**Affiliations:** Department of Veterinary Medicine and Surgery, College of Veterinary Medicine, University of Missouri, Columbia, MO, United States

**Keywords:** kittens, growth, sex, 3-epi-25-hydroxyvitamin D, 24,25-dihydroxyvitamin D, calcitriol

## Abstract

**Background:**

Vitamin D deficiency and excess in clinically presented cats conventionally is diagnosed by comparison of patient plasma 25-hydroxyvitamin D (25 (OH)D) concentration with plasma reference intervals determined in healthy adult cats. For immature cats, validity of this vitamin D status assessment method is uncertain.

**Objective:**

The overall objective was determination of whether plasma concentration of 25 (OH) D and other vitamin D metabolites in immature cats markedly change with developmental age as has been reported in other species.

**Methods:**

Four male and 4 female domestic short-hair kittens from weaning were continuously presented a single nutritionally adequate growth-diet. Concentrations of 25 (OH) D and 24,25-dihydroxyvitamin D (24,25 (OH)_2_D), and calcitriol were quantified in plasma of jugular venous blood collected at 12, 15, 18, and 21 weeks and 1 year of age. Plasma was liquid and solid-phase extracted and fractionation by normal-phase HPLC, and 25 (OH) D and 24,25 OH)_2_D quantified by reverse-phase HPLC-UV and calcitriol by RIA.

**Results:**

Plasma 3-epi-25 (OH) D and 25 (OH) D concentrations increased (*p* < 0.001) with age so that by study end the concentrations rose by 1-and 2-fold, respectively. Concentrations of 3-epi-25 (OH) D relative to 25 (OH) D were 30% at 12 weeks and 20% at 1 year. Between ages 12 and 21 weeks, rises in 25 (OH) D concentration were positively correlated with body weight gains (*ρ* = 0.952, *p* < 0.001) and 24,25 (OH)_2_D concentrations were consistently greater than 25 (OH) D concentrations (*p* < 0.001). At 1 year of age, concentrations of 24,25 (OH)_2_D declined below those of 25 (OH) D and 3-epi-24,25 (OH)_2_D consistency occurred in low concentrations. Vitamin D_2_ metabolites and sex differences in metabolite concentrations were not observed.

**Conclusion:**

Reliance on quantification of plasma 25 (OH) D concentration for vitamin D status assessment in kittens may be confounded by developmental changes in 25 (OH) D independent of vitamin D intake. High 24,25 (OH)_2_D concentration and occurrence of 3-epi-25 (OH) D in plasma additionally may interfere with the quantification.

## 1 Introduction

Vitamin D status is conventionally assessed though measurement of plasma concentration of the metabolite, 25-hydroxyvitamin D (25 (OH)D). 25 (OH) D is readily measurable, has a long circulating half-life, and occurs in concentrations assumed to be proportional to body vitamin D stores. In recent years, quantification of 25 (OH) D in plasma in cats has become complicated by discovery of C-3 epimers of 25-hydroxyvitamin D_3_ (25 (OH)D_3_) and 25-hydroxyvitamin D_2_ (1, 2). Because of recommendations for supplementing feline diets with vitamin D_3_ (cholecalciferol) rather than vitamin D_2_ (ergocalciferol) (3), finding 25-hydroxyvitamin D_2_ epimers in cat plasma should be rare. The C-3, α-epimeric form of 25 (OH)D_3_ is occurs in concentrations of 10 to 50% of concentrations of the β-epimeric form of 25 (OH)D_3_ (4). The more abundant 25 (OH)D_3_ β-epimer readily serves as substrate for synthesis of the calciotropic hormone derivative of vitamin D, calcitriol or 1α,25-dihydroxyvitamin D (5). An α-epimeric form of calcitriol has been identified and investigated *in vitro* (6), but it has not been reported in plasma or tissues of cats. If present in cats, α-epimeric calcitriol likely would have little calciotropic activity as *in vivo* and *in vivo* studies of tissues and animals of other species show that α-epimeric calcitriol has low functionality, except for one notable exception; α-epimeric calcitriol is nearly as potent as calcitriol for inhibiting PTH secretion by bovine parathyroid cells in primary cultures (7).

Typically, high-throughput, immunoassays developed to quantify 25 (OH) D in human serum are used for clinical diagnostic analysis of cat samples. Concentrations of 25 (OH) D determined with these methods may be inaccurate because of unknown cross-reactivities with plasma 3-epi-25 (OH)D_3_ and the dihydroxy vitamin D metabolite, 24,25-dihydroxyvitamin D (24,25 (OH)_2_D) (8, 9), which is abundant in cats. Concentrations of 24,25 (OH)_2_D in human plasma are typically low, only about 10% of concentrations of 25 (OH)D. Whereas in cat plasma, concentrations of 24,25 (OH)_2_D_3_ are 30 to 50% of those of 25 (OH)D_3_ (2, 10, 11). Additionally, C-3 epimers of 24,25 (OH)_2_D_3_ are reported in cat plasma, with the α-epimeric form of 24,25 (OH)_2_D_3_ occurring at low concentrations relative to the β-epimeric form (4).

The 24,25 (OH)_2_D in plasma samples may be extracted before 25 (OH) D determinations to lessen immunoassay or other competitive-binding assay interference (12). However, extraction of human samples is likely not necessary for clinical diagnostic purposes because 24,25 (OH)_2_D concentrations are low in plasma and vitamin D intake only marginally affects the ratio of 24,25 (OH)_2_D to 25 (OH) D (13). Therefore, clinical interpretation of 25 (OH) D immunoassay results of human compared to cat samples is potentially less affected by cross-reactive 24,25 (OH)_2_D.

Developmental changes in vitamin D metabolism may also confound reliance on plasma 25 (OH) D concentration for assessment of vitamin D status. Problematic for determining vitamin D status in children is variable abundance of 3-epi-25 (OH) D in their plasma (14). The α-epimeric 25 (OH) D concentrations range from 9 to 60% of the total concentration of 25 (OH) D, and they decline rapidly with age during the first year of life. Plasma vitamin D metabolite concentrations in immature dogs have been described (15). Reported findings precede availability of methods required for quantitation of C-3 epimers of the metabolites. Nevertheless, large changes in plasma concentrations of 25 (OH) D, 24,25 (OH)_2_D, and calcitriol occur in puppies as they mature (16). These changes are important to note as they are independent of dietary vitamin D concentration and greatly influenced by breed size and rate of body weight gain.

There are few reports on plasma vitamin D metabolite concentrations in immature cats. For kittens between ages of 9 and 22 weeks, Morris et al. (17) showed that plasma 25 (OH) D concentrations increase with age and dietary vitamin D content. Cause for the change with age was not clear because the kittens were initially depleted of vitamin D. The observed increase in plasma 25 (OH) D concentration with age may have reflected developmental changes in vitamin D metabolism or simply a long plasma equilibration time for 25 (OH)D. Rise in plasma 25 (OH) D to a plateau concentration occurs only after 8 weeks or more in human adults daily taking oral vitamin D supplements (18). Pineda et al. (19) quantified with immunoassay methods two plasma vitamin D metabolites in kittens that received a commercial growth diet containing an adequate and consistent amount of vitamin D. The investigators found that plasma 25 (OH) D concentrations at 12 weeks of age were about half the values observed at 12-and 15-months age. They also found that plasma calcitriol concentrations were greater at 12 weeks than at 12-and 15-months of age.

The overall objective of the present research was to quantify with chromatographic methods the plasma concentrations of 25 (OH) D, 24,25 (OH)D_2_, and their C-3 enantiomers, and calcitriol in kittens fed a diet with adequate and unchanging vitamin D content. The purpose of the research was to investigate whether developmental age should be considered when using plasma 25 (OH) D concentration for identifying vitamin D deficiency and excess in kittens. Age-related changes in the vitamin D metabolite abundances were hypothesized to occur as the kittens matured.

## 2 Materials and methods

### 2.1 Animals

Four male and 4 female, university-owned, purpose-bred, domestic shorthair kittens born during the summer months of 2021 were studied. The kittens were derived from four litters. Dams or sires of the kittens were heterozygous carriers of an autosomal dominant mutation of the polycystin gene which causes polycystic kidney disease (20). Genotyping of the kittens showed them to be negative for the mutation. Physical examination and clinical hematology and chemistry evaluations revealed that the kittens had no abnormalities. The kittens were segregated by sex following weaning and were group-housed in a humidity, temperature and light-cycle controlled facility accredited by American Association for Laboratory Animal Science. The kittens were *ad lib* fed and watered. Body weights were recorded weekly. Husbandry and treatment of the kittens were in accordance with the Guide for the Care and Use of Laboratory Animals and reviewed and approved by the Animal Care and Use Committee of the University of Missouri (protocol#20460). The kittens were neutered and adopted as pet companions when they were between 21 and 42 weeks of age.

### 2.2 Diet

Multiple manufacturer batches of a commercially available, dry-expanded diet were presented during the study (Royal Canin Feline Health Nutrition Kitten, St. Charles, MO, United States). As per the manufacturer, the diet was formulated to meet the nutritional levels established by the Associated of American Feed Control Officials for feline growth. Samples of five batches of diet presented during the study were combined in equal portions and submitted for moisture and vitamin D analysis by a commercial laboratory (Eurofins Nutrition Analysis Center, Des Moines, IA, United States). Analyses results and nutrient composition of the diet is listed in Supplementary Table S1.

### 2.3 Design

Samples of blood (3-5 mL) were collected from the kittens at 12, 15, 18, and 21 weeks of age and once after adoption when they were between 52 to 54 weeks of age. Adoptive owners were informed of value and risks of the research during an interview, after which they acknowledged by written consent agreement to participate. They were further instructed to feed only provided study diet until a time scheduled of blood collection. The kittens were briefly (< 5 min) confined in a restraint bag for blood collection by jugular venipuncture with a 23-gauge needle. Sampled blood was added to tubes containing lithium heparin (~19 USP units/mL) and plasma thereafter extracted within 30 min of collection by centrifugation for 10 min at 1,200 × g. The plasma was stored frozen at -80°C until vitamin D metabolite determinations.

### 2.4 Laboratory determinations

Concentrations of 25-hydroxyvitamin D_3_ (25 (OH)D_3_) and 24,25-dihydroxyvitamin D_3_ (24,25 (OH)_2_D_3_) and those of their C-3 epimers in plasma were determined using previously described chromatographic methods with minor modifications for determining metabolite recoveries (2). In place of radiolabeled internal standards, the α-epimer of 25-hydroxyvitamin D_2_ (3-epi-25-hydroxyvitamin D_2_) and 24*RS*,25-dihydroxyvitamin D_2_ (Isosciences, Ambler, PA) were added (25 or 50 ng in 20 μL of methanol) and incubated overnight with assayed plasma aliquots of 0.5 or 1.0 mL at 4°C, before liquid and solid-phase extractions. Extraction efficiencies of the vitamin D_2_ metabolites were not different from those of the quantified vitamin D_3_ metabolites. No detectable UV peaks of absorbance at 265 nm were found to elute at retention times of the internal standards in plasma samples not spiked with the internal standards. This observation was consistent with vitamin D_2_ not being detected in the study diet (< 0.1 μg/kg).

Concentrations of calcitriol in plasma were determined using a commercial RIA kit (AA-54, Immunodiagnostic Systems, Gaithersburg, MD) as previously described but with a few modifications (2). In brief, to remove interferences that might affect the calcitriol RIA, plasma aliquots (0.5 mL) were extracted and HPLC fractionated by the methods used in quantification of the other hydroxy-vitamin D metabolites. Losses of calcitriol were estimated from recovery of 3-epi-25-hydroxyvitamin D_2_ rather than tritiated calcitriol added to plasma samples. Use of 3-epi-25-hydroxyvitamin D_2_ for this purpose was assessed from comparisons of recoveries of 3-epi-25-hydroxyvitamin D_2_ with those of calcitriol. For this, 0.5 mL plasma replicates (*n* = 6) were mixed and incubated overnight at 4°C with a 20 μL methanolic spike containing 50 ng of 3-epi-25-hydroxyvitamin D_2_ and 50 ng of 1α,25-dihydroxyvitamin D_3_. Spike recoveries after extraction and fractionation by the assay methods were determined by HPLC.

Prior to plasma calcitriol determinations, recoveries of from the six plasma spikes of 1α,25-dihydroxyvitamin D_3_ compared to 3-epi-25- (OH)D_2_ were found to be consistent, but less by a mean (± SD) of 59% (± 7%). Hence, sample calcitriol recovery was as taken to be 59% of observed 3-epi-25-hydroxyvitamin D_2_ recovery. Mean (± SD) recovery of 3-epi-25-hydroxyvitamin D_2_ across all samples analyzed for calcitriol content was 59% (± 10%).

### 2.5 Statistical analysis

Significance of age and sex effects on body weights and concentrations of the vitamin D metabolites and their ratios were assessed using repeated-measures, mixed-model ANOVA. For this, fixed effects were age, sex, and age-sex interaction and kitten subject was assigned to be a random effect. Tukey-adjusted, multiple comparisons were used to determine significance of age differences. Variable observations were considered normal when means and medians of observations were within 10% of each other and skew and excess kurtosis of observations each were between −1 and 1. Body weight and 25 (OH)D_3_ concentration observations were normally distributed after logarithmic transformation.

At study end, significance of sex difference in 3-epi-24,25- (OH)_2_D_3_ concentrations was determined with a two-sample t-test. For the period between 12 and 21 weeks of age, significance of effect of sex on percentage change in body weight was determined with a Wilcoxon-sign test, and significance of correlation between percentage change in body weight and increase in 25 (OH)D_3_ concentration was determined with a Spearmen-rank correlation test.

Statistical analyses were conducted with computer software (SAS 9.4, SAS Institute, Cary, NC, United States). Variable observations are reported as means and variances expressed as SEM. Effects were considered significant if *p* < 0.05.

## 3 Results

Body weights at 12 weeks did not significantly differ by sex (Table 1). After the kittens were 15 weeks of age and until the study end, body weights were greater (*p* < 0.001) for males than females. Males gained body weight at a more rapid rate than females. From 12 and 21 weeks of age, the increase in body weight of males was about 30% greater (*p* = 0.021) than that of the females (median (range): 113 (99–128)% vs. 80 (72–86)%). When mature body weights were determined, median body weights compared to 12 weeks of age for males more than tripled while those for females approximately doubled.

**Table 1:** Body weights of male (*n* = 4) and female (*n* = 4) kittens from 12 weeks to 1 year of age.

		Age^1^						*p*-value		
		12 weeks	15 weeks	18 weeks	21 weeks	1 year^2^	SE	Age	Sex	Age × Sex
Body weight^3^										
kg	All	1.58^a^ (1.32–1.83)	2.08^b^ (1.68–2.50)	2.55^c^ (2.05–3.26)	2.96^d^ (2.33–3.83)	4.58^e^ (3.41–6.15)	1.04	< 0.001	< 0.001	0.004
	Males	1.76^a^ (1.69–1.83)	2.32^b^ (1.90–2.50)	2.88^c^ (2.30–3.26)	3.45^d^ (2.91–3.83)	5.77^e^ (5.45–6.15)	1.04			
	Females	1.42^a^ (1.32–1.55)	1.87^b^ (1.68–2.08)	2.25^c^* (2.05–2.52)	2.55^c^* (2.33–2.77)	3.63^d^* (3.41–4.17)	1.04			
%initial^4^	All	100^a^	132^b^ (108–145)	161^c^ (131–186)	188^d^ (165–219)	271^e^ (202–364)	1.03	< 0.001	0.014	< 0.001
	Males	100^a^	132^b^ (128–137)	164^c^ (151–163)	196^d^ (172–186)	341^e^ (202–247)	1.03			
	Females	100^a^	132^b^ (108–145)	158^c^ (131–186)	180^c^ (165–219)	215^d^* (322–364)	1.03			

Data are mean (range) observations on four male and four female kittens. *Significantly different from observations in males. ^1^Where letter annotations in a row are not the same, differences between observations were significant (*p* < 0.05). ^2^52 to 54 weeks of age. ^3^Observations log-transformed before statistical analyses. ^4^Percentage relative to body weight at 12 weeks of age. SE = standard error.

**Figure 1:**
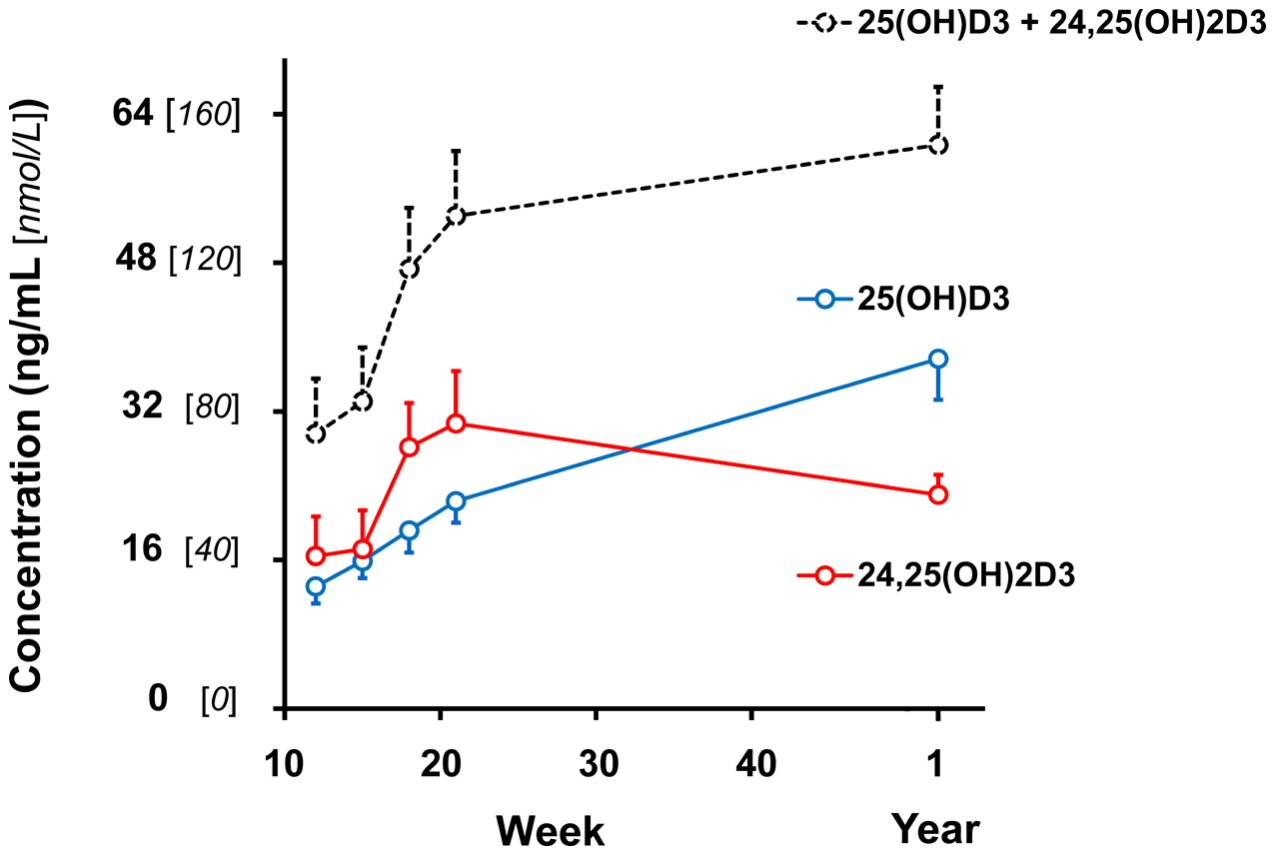
Plasma vitamin D hydroxy-metabolite concentrations of male and female kittens when ages of 12, 15, 18, and 21 weeks and 1 year (52-54 weeks). Plot points are mean values of the sum of α- and β-epimer concentrations observed for the metabolites. Associated vertical bars represent SEM calculated from 8 Plasma vitamin D hydroxy-metabolite concentrations of male and female kittens when ages of 12, 15, 18, and 21 weeks and 1 year (52-54 weeks). Plot points are mean values of the sum of α- and β-epimer concentrations observed for the metabolites. Associated vertical bars represent SEM calculated from 8 observations.

**Figure 2:**
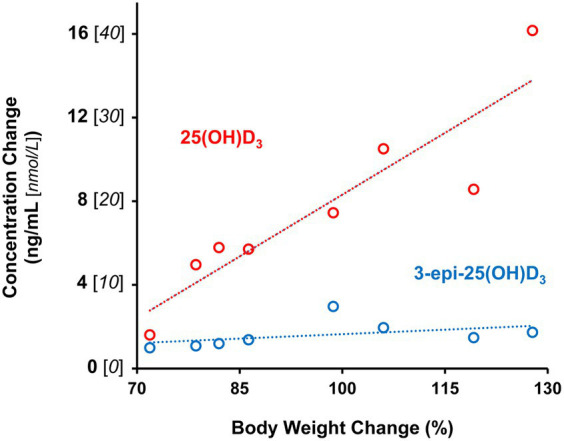
Age-associated plasma 25-hydroxyvitamin D metabolite increases are correlated with percentage change in body weight of male and female kittens between 12 and 21 weeks of age. Dotted lines are plots linear functions of derived from observations for each metabolite (*n*=8).

Plasma 25 (OH)D_3_ concentrations were not significantly different between males and females at 12 weeks of age (Table 2). As the kittens matured, plasma 25 (OH)D_3_ concentrations continuously increased (Figure 1) but varied with rate of body weight gain. Plasma 25 (OH)D_3_ concentrations were positively correlated (*ρ* = 0.952, *p* < 0.001) with increase in percentage body weight between weeks 12 and 21 (Figure 2). At maturity (52-54 weeks of age), plasma 25 (OH)D_3_ concentrations for all kittens were greater (*p* < 0.001) than concentrations at weeks 12, 15, 18, and 21 by approximately 170, 130, 90, and 60%, respectively. An adoptive owner of one kitten, a female, elected to give another dry-expanded diet that met recommendations for feline growth. The kitten received the diet continuously for 12 weeks before its last blood collection when the kitten weighed 4.17 kg and plasma 25 (OH)D_3_ and 3-epi-25-(OH)D_3_ concentrations were 24.6 and 9.3 ng/mL, respectively.

**Table 2:** Plasma concentrations of vitamin D metabolites in male (*n* = 4) and female (*n* = 4) kittens from 12 weeks to 1 year of age.

		Age^1^						*p*-value		
		12 weeks	15 weeks	18 weeks	21 weeks	1 year	SE	Age	Sex	Age × Sex
Metabolite concentration										
25(OH)D_3_^2^ (ng/mL)	All	9.2^a^ (5.5–21.3)	11.2^a^ (7.6–23.8)	13.9^ab^ (7.6–29.1)	16.8^b^ (11.0–27.1)	28.4^c^ (15.8–48.1)	1.2	< 0.001	0.843	< 0.300
	Males	7.8^a^ (5.5–10.4)	10.2^b^ (7.6–13.3)	13.9^c^ (9.8–18.2)	18.1^c^ (12.9–26.6)	30.0^d^ (20.4–45.9)	1.2			
	Females	10.9^a^ (6.0–21.3)	12.3^ab^ (7.8–23.8)	14.0^ab^ (7.6–29.1)	15.6^b^ (11.0–27.1)	26.9^c^ (15.8–48.1)	1.2			
3-epi-25-(OH)D_3_^3^ (ng/mL)	All	3.1^a^ (1.9–4.1)	3.9^ab^ (2.4–4.7)	4.2^b^ (2.7–5.5)	4.7^b^ (2.9–6.2)	7.3^c^ (5.7–9.3)	0.3	< 0.001	0.179	0.195
	Males	3.4 (3.0–4.1)	4.0 (3.0–4.7)	4.7 (3.9–5.5)	5.4 (4.5–6.2)	7.2 (6.5–8.8)	0.3			
	Females	2.8 (1.9–3.8)	3.8 (2.4–4.5)	3.7 (2.7–4.3)	4.0 (2.9–5.0)	7.4 (5.7–9.3)	0.3			
24,25(OH)_2_D_3_ (ng/mL)	All	16.4^a^ (6.1–41.2)	17.0^a^ (6.2–41.3)	27.5^abc^ (7.8–44.7)	30.2^c^ (10.9–55.7)	20.7^abc^ (14.6–32.4)	4.5	0.003	0.707	0.356
	Males	16.0 (6.1–24.4)	11.9 (6.2–17.6)	24.8 (7.8–36.5)	32.9 (10.9–55.7)	18.9 (14.6–25.9)	5.4			
	Females	16.9 (7.8–41.2)	22.1 (8.3–41.3)	30.2 (13.5–44.7)	27.4 (18.4–51.1)	22.4 (17.1–32.4)	5.4			
1,25(OH)_2_D_3_^2^ (pg/mL)	All	122^abc^ (56–304)	108^ac^ (44–250)	153^b^ (87–340)	155^ab^ (84–368)	107^c^ (42–241)	26	0.005	0.519	< 0.001
	Males	147^ab^ (79–304)	130^ab^ (86–250)	138^ab^ (87–340)	217^a^ (149–368)	85^b^ (42–155)	34			
	Females	102^ab^ (56–150)	90^a^ (44–157)	170^b^ (135–201)	111^ab^ (84–186)	135^a^ (100–241)	34			
Metabolite concentration ratio to 25(OH)D3 concentration										
3-epi-25-(OH)D_3_^3^	All	0.36^ab^ (0.18–0.61)	0.38^a^ (0.16–0.58)	0.32^abc^ (0.15–0.56)	0.29^bc^ (0.18–0.48)	0.27^c^ (0.17–0.41)	0.05	0.002	0.425	0.008
	Males	0.46^a^ (0.32–0.61)	0.41^ab^ (0.23–0.57)	0.36^abc^ (0.22–0.56)	0.32^bc^ (0.19–0.48)	0.25^c^ (0.18–0.32)	0.06			
	Females	0.27 (0.18–0.48)	0.33 (0.16–0.58)	0.28 (0.15–0.45)	0.26 (0.18–0.36)	0.29 (0.17–0.41)	0.06			
24,25(OH)_2_D_3_^2^	All	1.3^a^ (0.8–2.3)	1.2^a^ (0.7–2.4)	1.5^a^ (0.6–3.3)	1.4^a^ (0.6–4.3)	0.7^b^ (0.5–1.3)	1.2	< 0.001	0.665	0.211
	Males	1.6 (0.9–2.3)	1.0 (0.7–1.4)	1.3 (0.6–2.6)	1.3 (0.6–4.3)	0.6 (0.5–0.7)	1.2			
	Females	1.1 (0.8–1.9)	1.4 (1.1–2.4)	1.9 (1.5–3.3)	1.6 (1.2–1.9)	0.8 (0.7–1.3)	1.2			
1,25(OH)_2_D_3_^2^ (x 100)^4^	All	1.3^a^ (0.7–4.6)	1.0^a^ (0.6–3.0)	1.1^a^ (0.6–2.6)	0.9^a^ (0.7–2.1)	0.4^b^ (0.2–0.8)	0.3	< 0.001	0.559	0.003
	Males	1.9^a^ (0.8–4.6)	1.3^a^ (0.6–3.0)	1.0^a^ (0.6–2.6)	1.1^a^ (0.7–2.1)	0.3^b^ (0.2–08)	0.4			
	Females	0.9^ab^ (0.7–1.1)	0.7^ab^ (0.6–1.0)	1.2^b^ (0.7–2.4)	0.7^ab^ (0.7–0.8)	0.5^b^ (0.4–06)	0.5			

Data are mean (range). ^1^Where letter annotations in a row are not the same, differences between observations were significant (*p* < 0.05). ^2^Observations log-transformed before statistical analyses. ^3^C-3, α-epimer of 25(OH)D_3_. ^4^Ratios of concentrations of 1,25(OH)_2_D_3_ to 25(OH)D_3_ multiplied by 100 to facilitate visual comparisons of data. SE = standard error; 25(OH)D_3_ = 25-hydroxyvitamin D_3_; 24,25(OH)_2_D_3_ = 24,25-dihydroxyvitamin D_3_; 1,25(OH)_2_D_3_ = 1α,25-dihydroxyvitamin D_3_ or calcitriol.

Concentrations of 3-epi-25-(OH)D_3_ in all sampled plasma did not significantly differ between males and females and they were always less (*p* < 0.001) than its enantiomer, 25 (OH)D_3_ (Table 2). Concentrations of 3-epi-25-(OH)D_3_ increased with age (*p* < 0.001) but with differing proportion relative to 25 (OH)D_3_ concentrations. The 3-epi-25-(OH)D_3_ concentrations at 12 weeks of age were about 30% of the total (α + β epimer) of 25 (OH)D_3_ concentrations. When mature at 52-54 weeks, the 3-epi-25-(OH)D_3_ proportion was about 20% of the total of 25 (OH)D_3_ concentrations. The increase in plasma 3-epi-25- (OH)D_3_ concentrations between 12 to 21 weeks of age was small but correlated (*ρ* = 0.786, *p* < 0.021) with increase in percentage body weight (Figure 2).

Concentrations of 24,25 (OH)_2_D_3_ in plasma substantively changed with age but were not significantly different between males and females (Table 2). From 12 to 21 weeks, median 24,25 (OH)_2_D_3_ concentrations among the kittens nearly doubled (Figure 1) and overall concentrations of 24,25 (OH)_2_D_3_ were greater than those of 25 (OH)D_3_ (*p* < 0.001). The 24,25 (OH)_2_D_3_ concentrations at maturity (52-54 weeks) markedly varied so that across all cats the concentrations were not significantly different from 24,25 (OH)_2_D_3_ concentrations of the prior period of growth, weeks 12 to 21. The median ratio of 24,25 (OH)_2_D_3_ to 25 (OH)D_3_ concentration at 52-54 weeks inverted so that median 24,25 (OH)_2_D_3_ concentration declined to about two-thirds of the median of 25 (OH)D_3_ concentration. The C-3, α-epimer of 24,25 (OH)_2_D_3_ was inconsistently detected in samples collected during weeks 12, 15, 18, and 21. In contrast, this α-epimer was quantifiable in all plasma sampled at 52-54 weeks when its median (range) concentration across all cats was 2.2 (1.7-3.2) ng/mL. There was no significant difference in concentrations of the α-epimer of 24,25 (OH)_2_D_3_ with sex.

Plasma concentrations of calcitriol were not different between males and females but were significantly (*p* = 0.005) different with age (Table 2). Calcitriol concentrations among the kittens at week 18 of age were greater than when they were 1 year of age (*p* = 0.041). The ratio of plasma calcitriol concentration to 25 (OH)D_3_ concentration at 1 year of age (1: 244) was much less (*p* < 0.001) than ratios when the kittens were 12 to 21 weeks of age (1, 75 to 1: 114).

## 4 Discussion

The focus of the present study was quantification of 25 (OH)D_3_ in plasma of a cohort of post-weaned kittens given a single diet during the first year of life. The study diet contained 1,290 IU/kg of vitamin D3 on a dry matter (DM) basis (7.9 μg/Mcal). This amount more than exceeded the dietary allowance for feline growth (224 IU/kg DM or 1.4 μg/Mcal) but was well less than the recommended safe upper limit (30,000 IU/kg DM or 188 μg/Mcal) (3). Observed 25 (OH)D_3_ concentrations changed greatly during the 9-week period of growth studied. Body weights of the kittens during the period more than doubled in females and tripled in males. The 25 (OH)D_3_ concentrations increased linearly with time by more than 1.5-fold in females and 2.5-fold in males. Although 25 (OH)D_3_ concentrations greatly rose, they were significantly less than 25 (OH)D_3_ concentrations observed at 1 year of age for both sexes. This trend with age agreed with findings of Pineda et al. (19) who gave kittens a single growth diet that contained vitamin D (1,500 IU/kg) in a concentration similar to the diet presently used. Sih et al. (21) also observed an increase in plasma 25 (OH)D_3_ concentrations of kittens with age, even though their kittens were raised on a diet of extraordinarily high dietary vitamin D_3_ content – 33,840 IU/kg. Plasma 25 (OH)D_3_ concentrations of the kittens at 3 months of age increased with time so that by 18 of months of age they were 4-fold greater. Rising plasma 25 (OH)D_3_ concentration during growth has been reported for miniature poodle puppies when they are given a constant dietary vitamin D content (16). Interestingly, the body weights and growth rates of the puppies were similar to those observed in kittens in the present study.

The cause for increasing plasma 25 (OH)D_3_ concentration with age is not apparent. Plasma concentration of 25 (OH)D_3_ and other vitamin D metabolites rise until metabolite entry rate is matched by clearance rate. With respect to entry, most circulating 25 (OH) D derives from liver where 25-hydroxylation of available vitamin D occurs principally though activities of the cytochrome P450 enzymes, CYP2R1 and CYP27A1 (22). Increasing activity of these enzymes might have accounted for the strongly positive correlation found between body weight gain of the kittens and their plasma 25 (OH)D_3_ concentrations. When nutrition is adequate, rate of growth is controlled by an axis of growth hormone (GH) and insulin-like growth factor (IGF-1). Past investigations report that IGF-1 concentrations increase in plasma of kittens as they grow (23, 24). This trend is believed to be secondary to action of rising GH concentration on liver IGF-1 production. Hormonal regulation studies of human hepatoblastoma cells have shown that both GH and IGF-1 stimulate expression and activity of CYP27A1 (25). If GH and IGF-1 stimulate liver 25-hydroxylation *in vivo*, an increasing production of 25 (OH) D from rising liver 25-hydroxylase activity might explain the presently observed relationships between age, rate of growth, and plasma 25 (OH) D concentration. Supportive of this speculation are findings of low circulating 25 (OH) D concentrations in GH-deficient children and normalization of the 25 (OH) D concentrations following GH treatment (26). Additionally, positive correlation between plasma IGF-1 and 25 (OH) D concentrations has been reported in some but not all cohorts of children (27).

The C-3 epimer, 3-epi-25-(OH)D_3_, was found in plasma of all the kittens but not in extraordinarily high concentrations as reported in human infants (14). The epimeric metabolite’s concentrations were highest at 1 year of age, yet for all ages they were less than 25 (OH)D_3_ concentrations, as has been reported in adult cats (1, 4, 11, 28). Like the trend with age observed for plasma 25 (OH)D_3_, 3-epi-25-(OH)D_3_ concentrations increased with body weight gain during the studied 9-week period of growth. The rise in plasma 3-epi-25-OH)D_3_ was less than that of 25 (OH)D_3_. A mechanistic cause for the change with age and its relationship to gain in body weight was not evident. Production of C-3 epimers occurs by pathways independent of 25-hydroxylation and varies across tissues and cell types (6). The epimerization was first discovered in cultured human keratinocytes (29). Later, *in vivo* studies of mouse keratinocyte carcinoma cells revealed that a substantive amount of 3-epi-25-(OH)D_3_ occurs in skin (30). This led to speculation by investigators that a significant portion of circulating 3-epi-25-(OH)D_3_ may be derived from skin. In considering this, the presently observed relationship between body weight gain of the kittens and rise in plasma 3-epi-25-(OH)D_3_ concentration might be explained. An increasing plasma entry of 3-epi-25-(OH)D_3_ might be derived from keratinocytes of an enlarging mass of skin during growth. The lesser increase in plasma 3-epi-25-(OH)D_3_ relative to 25 (OH)D_3_ might reflect growth rate differences of tissues producing the metabolites, namely skin and liver, respectively. Enlargement of the skin mass should lag behind that of the liver mass because skin surface area scales with body weight to the 0.67 power (31), whereas liver cytochrome P450 content (which includes CYP2R1 and CYP27A1) scales with body weight to the 0.95 power (32).

Accounts of 24,25 (OH)_2_D_3_ in plasma of cats are limited to reports on mature cats, where 24,25 (OH)_2_D_3_ concentrations relative to 25 (OH)D3 concentrations are lower but readily quantifiable (4, 10, 11, 33). Plasma 24,25 (OH)_2_D_3_ concentrations observed in kittens of the present study were always greater than 25 (OH)D_3_ concentrations, and they increased between 12 and 21 weeks of age by approximately 1-fold. Remarkably, a similar trend of rising plasma 24,25 (OH)_2_D_3_ concentrations relative to lower 25 (OH)D_3_ concentrations has been described in growing puppies (16). The apparent parallel increases in plasma concentrations 24,25 (OH)_2_D_3_ and 25 (OH)D_3_ may have reflected a substrate-product relationship between the metabolites. The cytochrome P450 enzyme, CYP24A1, which is found in kidney and many other tissues, catalyzes 24-hydroxylation of 25 (OH)D_3_ to form 24,25 (OH)_2_D_3_ (34). Therefore, with increasing 25 (OH)D_3_ concentration, greater 24,25 (OH)_2_D_3_ concentration should result. Indeed, observations in mature cats are consistent with this substrate-product relationship. Plasma concentrations of 24,25 (OH)_2_D_3_ in cats of 9 to 13 years in age rise as their plasma concentrations of 25 (OH)D_3_ increase following dietary 25 (OH)D_3_ supplementation (4). Some investigators have postulated that 24,25 (OH)_2_D_3_ has functional roles in cartilage maturation and prevention of skeletal abnormalities during rapid growth (15). Other investigators refute such attribution to 24,25 (OH)_2_D_3_ (5). Rising plasma 24,25 (OH)_2_D_3_ may only be of importance as an indicator of CYP24A1 activity, which reduces 25 (OH)D_3_ available for calcitriol synthesis and inactivates calcitriol by 24-hydroxylating it to form 1α,24,25-trihydroxyvitamin D. Without adequate CYP24A1 activity vitamin D toxicosis may occur (35). When CYP24A1 activity is marginal or lacking in humans a detrimental hypercalcemia occurs with coincidently low 24,25 (OH)_2_D_3_ and high 25 (OH)D_3_ concentrations in plasma (34).

Plasma concentrations of calcitriol observed did not vary as greatly as those of 25 (OH) D or 24,25 (OH)_2_D. Presumably this is because plasma ionized calcium concentration is tightly regulated and calcitriol relative to other vitamin D metabolites is much more potent in mobilizing calcium (36). Nevertheless, plasma concentrations of calcitriol observed in the kittens substantively changed. They were about 1-fold greater at 18 and 21 weeks of age than at 1 year of age. Pineda et al. (19) found a similar trend where plasma calcitriol concentrations of their kittens at 3 and 6 months of age were about 1.6-fold greater than concentrations at 12 months. Higher plasma calcitriol concentrations during growth compared to maturity may reflect a calcium homeostatic response to sequestration of circulating calcium for bone mineralization. Changes in plasma calcitriol with maturation also may reflect altered calcitriol entry secondary to age-dependent changes in GH secretion. As aforementioned, plasma GH concentrations of kittens increase during their growth and then later wane (23, 24). Calcitriol synthesis is stimulated by GH through its effect on activity of the kidney cytochrome P450 enzyme, CYP27B1, which 1α-hydroxylates 25 (OH) D to form calcitriol (37). This effect of GH is evident in acromegalic humans, where plasma concentrations of calcitriol and ionized calcium are elevated and then corrected with bromocriptine treatment (37). An effect of GH on calcitriol was also observed in puppies when calcitriol concentration in plasma and CYP27B1 expression in the kidneys greatly elevated in response to subcutaneous GH administration (38).

Changes in calcitriol abundance in plasma may also have contributed to the drop in the ratio of 24,25 (OH)_2_D_3_ to 25 (OH)D_3_ in plasma observed when the kittens matured. Studies on effectors of calcitriol concentration show that calcitriol stimulates kidney tubular CYP24A1 activity, which in turn feeds back to inactivate calcitriol by 24-hydroxylation (34). By this mechanism, rising plasma calcitriol expectedly would increase synthesis of 24,25 (OH)_2_D from 25 (OH)D. Hence, the high ratio of 24,25 (OH)_2_D_3_ to 25 (OH)D_3_ in plasma observed in the kittens might have resulted from extraordinary 24,25 (OH)_2_D_3_ production secondary to calcitriol stimulation of CYP24A1 activity. After maturation of the kittens, a reduced production of 24,25 (OH)_2_D_3_ should have occurred because of diminished stimulation of CYP24A1 by calcitriol.

In total, the present findings indicate that use of plasma 25 (OH) D concentration as an indicator of vitamin D status in kittens is potentially confounded by developmental changes in 25 (OH) D concentration. While vitamin D intake by kittens clearly affects plasma 25 (OH) D concentration (17), age and rate of body weight gain also appear to impact concentration of the metabolite. The selected method for measurement of 25 (OH) D concentration is potentially confounding as well. Immunoassays available for quantifying 25 (OH) D in plasma and serum may not detect 3-epi-25-(OH) D and might have a diminished cross-reactivity for 25 (OH)D_2_ (9). Of particular concern is use of immunoassays on kitten samples that may have substantial 24,25 (OH)_2_D cross-reactivity. If cross-reactivity toward 24,25 (OH)_2_D is high, trends with developmental age might appear different. Exemplary of this may be a past report on 25 (OH) D concentrations in kittens in which of investigators used an RIA but did not describe its sensitivities to closely related vitamin D metabolites (19). The 25 (OH) D concentrations reported by the investigators were approximately twice those presently found in kittens, and they trended with age in concentrations near the sum of 25 (OH) D and 24,25 (OH)_2_D concentrations presently observed. Recent specifications provided by the manufacturer of the RIA (AA-35F1, v13, Immunodiagnostic systems, Gaithersburg, MD) relay that relative cross-reactivities for 25 (OH)D_3_, 25 (OH)D_2_, and 24,25 (OH)_2_D_3_ are 100, 75, and > 100%, respectively. Therefore, results of the RIA were likely biased by the high 24,25 (OH)_2_D content in kitten plasma.

Our research was limited in that a small cohort of subjects was studied, only one dietary concentration of vitamin D was presented, adiposity was not assessed, and group housing precluded food intake determinations. Hence, findings should be applied generally with caution. With a greater subject number, significant vitamin D metabolite differences with sex might have been found, with body weight gain differences possibly accounting for a sex disparity. Males presently were observed to grow faster than the females, which is a widely recognized trait of cats (39). Rapid gain in body weight results in increased adiposity of cats (40), which might in turn affect circulating 25 (OH) D of cats. Excess body fat clearly lowers 25 (OH) D concentration in humans but evidently not so in dogs (41). Another limitation of the research is that age-related trends in metabolite concentrations might vary with cat breed, especially given that kitten growth is faster in large than small cat breeds (42). Marked plasma vitamin D metabolite concentration differences are found between small-breed and large-breed puppies (16). An order of magnitude lower plasma concentration of 24,25 (OH)D_3_ in large-breed compared to small-breed puppies is reported. The difference is noteworthy because a vitamin D metabolite imbalance is suggested as contributing to pathogenesis of developmental bone disease of large-breed dogs (15). Investigation of age-related trends in vitamin D metabolite profile of large and rapidly growing cats is warranted.

In summary, the present findings support the hypothesis that developmental changes occur in vitamin D metabolite concentrations of kittens that are independent of dietary vitamin D content. The results indicate that clinical laboratory reference intervals for plasma or serum 25 (OH) D concentrations that are derived from observations on adult cats are not likely useful for diagnosis of vitamin D deficiency or excess in kittens. The findings are consistent with previous observations of high concentrations of 24,25 (OH)D_3_ in plasma of cats and therefore justify caution in reliance on results of 25 (OH) D immunoassays, especially when interference from 24,25 (OH)D_3_ is unknown or not disclosed.

## Data availability statement

The raw data supporting the conclusions of this article will be made available by the authors, without undue reservation.

## Ethics statement

The animal studies were approved by Animal Care and Use Committee of the University of Missouri (protocol#20460). The studies were conducted in accordance with the local legislation and institutional requirements. Written informed consent was obtained from the owners for the participation of their animals in this study.

## Author contributions

RB: Conceptualization, Data curation, Formal analysis, Funding acquisition, Investigation, Methodology, Project administration, Resources, Supervision, Writing – original draft. DU: Conceptualization, Data curation, Investigation, Writing – review & editing.
