# Supplementary material for: Age-dependent changes in plasma concentrations of 25-hydroxyvitamin D may complicate vitamin D status assessment of immature cats
Source: Front Vet Sci. 2024 May 2;11:1365204. doi: 10.3389/fvets.2024.1365204 (PMC11097665; doi:10.3389/fvets.2024.1365204)
Supplement: Supplementary file 1 [file Table_1.DOCX]

**Supplementary Materials Table 1.** Nutrient contents and ingredients of study diet^*^.

| **Nutrient** | **Diet Amount** | | **NRC Amount**^†^ |
| --- | --- | --- | --- |
| **Analysis results**^‡^ | **per kg** | **per Mcal** | **per Mcal** |
| Vitamin D2 (µg) | < 1.0 | < 0.3 | - |
| Vitamin D3 (µg) | 30.5 | 7.9 | 1.4 |
| Moisture (g) | 53 | 14 | - |
| **Proximates**^§^ |  |  |  |
| Protein (g) | 340 | 88 | 56 |
| Protein (%metabolizable energy) | - | 31 | 20^¶^ |
| Fat (g) | 170 | 44 | 23 |
| Fat (%metabolizable energy) | - | 37 | 19^¶^ |
| Carbohydrate (g) | 280 | 73 | - |
| Carbohydrate (%metabolizable energy) | - | 32 | - |
| Fiber (g) | 19 | 4.9 | - |
| Moisture (g) | 52 | 13.5 | - |
| **Essential amino acids**^‡^ |  |  |  |
| Tryptophan (g) | 3.3 | 0.9 | 0.4 |
| Threonine (g) | 13 | 3.4 | 1.6 |
| Isoleucine (g) | 13 | 3.4 | 1.4 |
| Leucine (g) | 28 | 7.3 | 3.2 |
| Methionine (g) | 7.3 | 1.9 | 1.1 |
| Methionine + Cystine (g) | 13 | 3.2 | 2.2 |
| Phenylalanine (g) | 14 | 3.8 | 1.3 |
| Phenylalanine + Tyrosine (g) | 26 | 6.9 | 4.8 |
| Valine (g) | 14 | 3.8 | 1.6 |
| Arginine (g) | 19 | 5.0 | 2.4 |
| Histidine (g) | 7.0 | 1.8 | 0.8 |
| Lysine (g) | 14 | 3.7 | 2.1 |
| **Macrominerals**^§^ |  |  |  |
| Calcium (g) | 11 | 2.9 | 2.0 |
| Phosphorus (g) | 10 | 2.7 | 1.8 |
| Potassium (g) | 7.5 | 2.0 | 1.0 |
| Magnesium (g) | 1.0 | 0.3 | 0.1 |
| Sodium (g) | 4.7 | 1.2 | 0.3 |
| **Trace elements**^§^ |  |  |  |
| Zinc (mg) | 184 | 48 | 18 |
| Iron (mg) | 217 | 56 | 20 |
| Manganese (mg) | 61 | 16 | 1.2 |
| Copper (mg) | 14 | 3.7 | 2.1 |
| Selenium (μg) | 500 | 100 | 75 |
| Iodine (mg) | 4.5 | 1.2 | 4.5 |
| **Essential fatty acids**^§^ |  |  |  |
| Linoleic acid (g) | 35 | 9.2 | 1.4 |
| Linolenic acid (g) | 2.2 | 0.6 | 0.05 |
| EPA + DHA (g) | 2.9 | 0.8 | 0.025 |
| **Vitamins**^§^ |  |  |  |
| Choline (g) | 3.1 | 0.8 | 0.6 |
| Thiamin (mg) | 14 | 3.6 | 1.4 |
| Riboflavin (mg) | 51 | 13 | 1.0 |
| Niacin (mg) | 185 | 48 | 10 |
| Pantothenic acid (mg) | 60 | 16 | 1.4 |
| Pyridoxine (mg) | 41 | 11 | 0.6 |
| Folate (mg) | 11 | 3.0 | 0.2 |
| Cobalamin (µg) | - | nr | 5.6 |
| Biotin (mg) | 3.0 | 0.8 | 0.02 |
| Vitamin K (µg) | - | nr | 250 |
| Vitamin A (RAE, µg) | 6500 | 1700 | 75 |
| Vitamin D (µg) | 754 | 4.9 | 1.4 |
| Vitamin E (mg) | 566 | 150 | 9.4 |

^*^Ingredients listed on package label: chicken by-product meal, brown rice, brewers rice, chicken fat, corn gluten meal, wheat gluten, corn, natural flavors, egg product, dried plain beet pulp, powdered cellulose, fish oil, vegetable oil, sodium silico-aluminate, grain distillers dried yeast, sodium pyrophosphate, potassium chloride, psyllium seed husk, calcium carbonate, fructooligosaccharides, hydrolyzed yeast, choline chloride, DL-alpha tocopherol, L-ascorby-2-polyphosphate, niacin supplement, biotin, riboflavin supplement, D-calcium pantothenate, pyridoxine hydrochloride, vitamin A acetate, thiamine mononitrate, vitamin B12 supplement, folic acid, vitamin D3 supplement, taurine, salt, zinc proteinate, zinc oxide, manganese proteinate, ferrous sulfate, manganous oxide, copper sulfate, calcium iodate, sodium selenite, copper proteinate, L-lysine, marigold extract (Tagetes erecta L.), magnesium oxide, carotene, rosemary extract, mixed tocopherols, citric acid.

**^†^**Recommended Allowances of the nutrient requirements for growth of kittens after weaning from Table 15-10 of the National Research Council, Nutrient Requirements of Dogs and Cats, National Academy Press, Washington, DC, 2006.

^‡^Per kg analyses results reported by Eurofins Nutrition Analysis Center, Des Moines, IA, USA. Per Mcal values calculated from per kg results using metabolizable energy density listed on package label of the diet, 3,847 kcal/kg.

^§^Per kg values calculated from per Mcal values reported by manufacturer assuming diet energy density of 3,847 kcal/kg.

^¶^Calculated from NRC recommended allowance values assuming the metabolizable energy content of protein and fat are 3.5 and 8.5 kcal/g.

nr = not reported by manufacturer.
